# The effect of emotion regulation on happiness and resilience of university students: The chain mediating role of learning motivation and target positioning

**DOI:** 10.3389/fpsyg.2022.1029655

**Published:** 2022-12-09

**Authors:** Xiaoxue Chen, Zejuan Huang, Wei Lin

**Affiliations:** ^1^School of Intelligent Medical, Chengdu University of Traditional Chinese Medicine, Chengdu, China; ^2^School of Basic Medicine, Chengdu University of Traditional Chinese Medicine, Chengdu, China; ^3^School of Management, Chengdu University of Traditional Chinese Medicine, Chengdu, China

**Keywords:** emotion regulation, interpersonal relationship, learning motivation, target positioning, happiness, resilience, structural equation model, chain mediating role

## Abstract

**Objective:**

To investigate the effect andmechanism among emotion regulation, relationship,happiness, learning motivation, target positioning, and resilience of university students.

**Method:**

A total of 904 university students in China were included in this cross-sectional survey from April to May this year. The self-administered questionnaires, including the adapted Mental Health Scale with a Healthy Personality Orientation for College Students, were used to construct structural equations to test the chain mediating effects of learning motivation and target positioning based on a multi-stage whole group sample of university students.

**Result:**

Emotion regulation indirectly affected happiness through the mediating effect of interpersonal relationship (Med = −0.387, *p* = 0.001). Learning motivation and target positioning play the chain mediating role in the effect of emotion regulation on happiness (Med = −0.307, *p* = 0.001) and resilience (Med = −0.275, *p* = 0.001).

**Conclusion:**

Emotion regulation indirectly affected happiness and resilience through the chain mediating effect of learning motivation and target positioning.

## Introduction

Happiness, popularly referred to well-being or subjective well-being (SWB), is the research area of philosophy and religion (Diener et al., 2018). Its definition refers to the overall evaluation of an individual’s quality of life according to their internal standards, including life satisfaction and positive and negative emotions (Diener et al., 2018). Resilience, also a popular research area, is defined as an ability to adapt well when individuals encounter stressful events in life, such as adversity (Biao et al., 2022). Happiness and resilience are closely related to individual physical and mental health. Their contributing factors, internal mechanisms, and interventions are essential to positive psychology.

Much research has been conducted to study what aspects would influence happiness and resilience, and emotion regulation is one of important aspects. In terms of happiness, emotion regulation, as an essential component of social and emotional ability (Zhi et al., 2021), is a basic capability to maintain physical and mental well-being (Martin et al., 2017; Haijuan et al., 2022). Emotion regulation can improve happiness by enhancing positive emotions (Quoidbach et al., 2015) as emotion is significant to happiness from the definition (Li et al., 2007). Many studies have proved that emotion regulation correlated with well-being (OECD, 2015; Strickhouser et al., 2017). Besides, emotional regulation significantly predicts happiness (Zhi et al., 2021). Cognitive emotion regulation has a positive effect on happiness (Kohn et al., 2014). Moreover, cognitive appraisal, one emotion regulation strategy, also affects individual happiness (Li et al., 2007).

Resilience also highly correlates with emotion regulation (Gross, 1998; Juzhe et al., 2013). Previous studies have found that people with high resilience adopt positive emotion regulation and implement good emotional regulation strategies to deal with negative emotions. Emotional regulation also well reflects resilience (Bonanno and Diminich, 2013; Caina et al., 2016). Individuals with a high level of emotion regulation utilize positive emotion regulation strategies, which can be used as a protective factor to stimulate internal resources to stabilize emotional state, develop positive factors, and improve the level of psychological resilience in challenges and adversity (Tugade and Fredrickson, 2007). Regulatory emotion Self-efficacy of Emotional Regulation not only directly affects psychological resilience (Yong and Zhenhong, 2013; Wenjiang et al., 2020), but also plays a mediating role in family harmony and psychological resilience (Qisheng and Dan, 2017). For early gastric cancer patients, strengthening the intervention of emotional regulation ability can improve psychological resilience (Lili, 2019). Additionally, cognitive appraisal, one of the emotion regulation strategies, predicts psychological resilience (Guolai et al., 2017; Sukui and Yue, 2022).

However, what is the internal mechanism of the effect of emotional regulation on happiness and resilience? Are there other factors that might mediate the influence of emotional regulation on happiness and resilience? What roles do interpersonal relationships, learning motivation, and target positioning play in the impact of emotional regulation on happiness and resilience? In this article, we built a structural equation model to explore the relationship of emotion regulation, learning motivation, target positioning, interpersonal relationship, happiness, and resilience on the basis of previous empirical research.

## Hypothesis development

Since the 1980s, researchers have paid more attention to the influence of targets on happiness (Brunstein, 1993; Kasser and Ryan, 1996). Emmons pointed out that having the meaningful purposes in life and moving toward them is a prerequisite for well-being. Diener proposed in the Telic Theory that, as an important reference standard of affective system, target can affect the level of happiness and predict well-being. It is found that the internal goals orientation is highly related to positive emotions, higher satisfaction, and well-being (Vansteenkiste et al., 2006), which also positively predict happiness (Jiejie et al., 2021). Besides, Individuals with a heightened sense of life purpose tend to have more robust subjective well-being (Hooker et al., 2017).

Although there were relatively few studies on the impact of target positioning on resilience, some studies proved that target positioning significantly predicts resilience (Sagone and Caroli, 2014; Biao et al., 2022). Purpose in life is a good protective factor for resilience, which is conducive to resilience reorganization (Biao et al., 2022). Existential psychology believes that individuals who experience the purpose and meaning of life are more likely to take a certain personal attitude toward incidents in the face of suffering, and are less likely to be stroke down (Schulenberg et al., 2008).

Learning motivation and target positioning, which are of great importance to university students, affect the learning outcomes and future planning. As a complex system, learning motivation contains a series of subsystems. Target positioning, belonging to the subsystem of learning motivation as one of the motivation variables, is closely correlated to learning motivation (Ling and Dejun, 2003).

Learning motivation is affected significantly by emotional regulation. As an active and indispensable variable in the structure of learning motivation, emotion is a necessary basis for forming and realizing learning motivation (Jianzhong and Xingyun, 1995). Thus, emotion plays an important role in learning motivation (Shilu and Youzhi, 2005). Therefore, emotion regulation can mobilize students’ inner will and improve learning motivation by regulating positive emotions (Barrett et al., 2001). Emotion regulation strategies can also enhance the level of learning motivation (Macher et al., 2013). In Ling’s study, during the COVID-19 period, emotions and emotion regulation significantly predicted online learning motivation due to the impact of the pandemic environment (Ling and Kuiliang, 2021). Hence, based on the previous arguments, this article proposed the following hypothesis:

*Hypothesis 1*: Learning motivation and target positioning played a chain mediating role in the effect of emotion regulation on happiness.

*Hypothesis 2*: Learning motivation and target positioning played a chain mediating role in the effect of emotion regulation on resilience.

Interpersonal relationship plays a critical role in university students’ mental health (Jiyuan and Zhongzeng, 2012). There are many factors that affect interpersonal relationships, one of which is emotion regulation. Some studies proved that emotion, which is important to interpersonal relationship (Garner, 2010), affects how individuals deal with interpersonal communication. Thus, how to manage emotions affects the state of interpersonal relationships. Campos (Campos and Camras, 2004) proposed that the rational emotion expression and the control of emotional experience played an essential role in forming interpersonal relationships.

In addition, interpersonal relationships affect well-being. Previous research has shown that interpersonal relationships reflect happiness (Ling et al., 2007; Feng and Man, 2012). In China, interpersonal relationships have been a stronger indicator of happiness (Hong and Siping, 2012). Few studies directly explored the relationship between emotional regulation and interpersonal relationship (Lisong et al., 2012). Interpersonal relationship is often used as the mediating variable in the research on the influencing factors and mechanism of happiness (Tse and Yip, 2009; Ling et al., 2013; Yangjun et al., 2017). Moreover, it is found that interpersonal relationship plays a mediating role in the effect of emotional regulation on happiness among college students (Ling et al., 2013). Hence, based on the abovementioned arguments, we proposed the following hypothesis:

*Hypothesis 3*: Interpersonal relationship mediating the influence of emotion regulation on happiness.

In sum, most previous studies only considered the direct impact of emotional regulation on happiness and resilience, or other factors that mediate the relationship between emotional regulation, happiness, and resilience. They paid little attention to the relationship between learning motivation, goal positioning, emotion regulation, interpersonal relationship, happiness, and resilience. Therefore, this study makes the following hypotheses: (1) Learning motivation and target positioning play a chain mediating role in the influence of emotional regulation on happiness; (2) Learning motivation and target positioning play a chain mediating role in the influence of emotional regulation on psychological resilience; and (3) Interpersonal relationship plays a mediating role in the influence of emotional regulation on happiness among university students.

## Materials and methods

### Sampling method and participants

This cross-sectional study used data among university students from April 2022 to May 2022, the aim of which was to investigate the influence of emotion regulation on happiness and resilience. Cluster sampling and simple random sampling were used in the data collection. A total of 904 valid questionnaires were returned with 1,000 questionnaires being distributed, and the effective response rate was 90.4%. SPSS25.0 software was used for descriptive statistics analysis and structural equation models were constructed and tested using AMOS23.0 software. The bias-corrected non-parametric percentile confidence interval Bootstrap method was used to examine the chain mediating effect of learning motivation and target positioning between emotion regulation and happiness, and resilience using 2000 replicate samples (Rui et al., 2013).

### Research tools

#### The self-administered questionnaire

The questionnaire was a self-designed questionnaire containing demographic variables.

#### The mental health scale with a healthy personality orientation for college students

The Mental Health Scale with a Healthy Personality Orientation for College Students developed by Cheng Ke was selected and adapted for studying better. The adapted scale is mainly used to measure the mental health of college students at the level of healthy personality orientation and consists of 27 questions divided into six dimensions: emotion regulation, interpersonal relationship, happiness, learning motivation, target positioning, and resilience. Interpersonal relationship mainly involves the problems of getting along with the opposite sex, dealing with interpersonal problems, communicating with strangers, and being respected by others. Emotion regulation includes the problems of emotion, external influence, and emotion control. Happiness involves the problem of satisfaction with the present situation, meaning of life, fulfillment in life, and the sense of control in life. Resilience involves the problem of the desire of challenge, recovering from setbacks, and coping with challenging. Learning motivation involves the problem of self-discipline, lack of motivation, procrastination, concentration, and perseverance. Target positioning includes the problem of graduation plans, future plans, and a sense of meaning in life. The higher the score on the dimension of emotion regulation and resilience, the higher the level of mental health. The higher the score on the dimension of interpersonal relationship, happiness, learning motivation, and target positioning, the lower the level of mental health. The scale has internal consistency reliability of 0.887. In this study, the Cronbach’s alpha measured by the adapted scale was 0.626.

### Statistical analysis

SPSS (Version 25, IBM, 2020) was used to conduct descriptive analysis, Lambda Correlation, Spearman’s Correlation, and Pearson’s Correlation. Amos Graphics (IBM, 2021) was used to test the hypothetic structural equation model.

#### Model construction and variable design

##### Principles of structural equation modeling

Structural equation model is a statistical method which uses linear equations to explain multiple statistic relationships between variables simultaneously based on the covariance matrix of variables. It contains two parts: the measurement model and the structural model. The matrix equation is:


(1) X=Λxξ+δ

(2) Y=Λyη+ε

(3) η=βη+Γξ+ζ

(1) The formula is the measurement equation and (2) the formula is the structural equation. X is a vector composed of exogenous indicators, Y is a vector composed of endogenous indicators, ξ is a vector of exogenous latent variables, η is a vector of endogenous latent variables, Λx is a coefficient matrix that reflects the strength of the relationship between exogenous observation variables and exogenous latent variables; Λy is a coefficient matrix reflecting the strength of the relationship between endogenous observed variables and endogenous latent variables, δ represents the measurement error of exogenous variables, ε represents the measurement error of the endogenous variable, β represents the coefficient matrix of the endogenous latent variable, Γ represents the coefficient matrix of the exogenous latent variable, and ζ represents the error of the structural equation.

##### Structural equation model and index design

**Figure 1:**
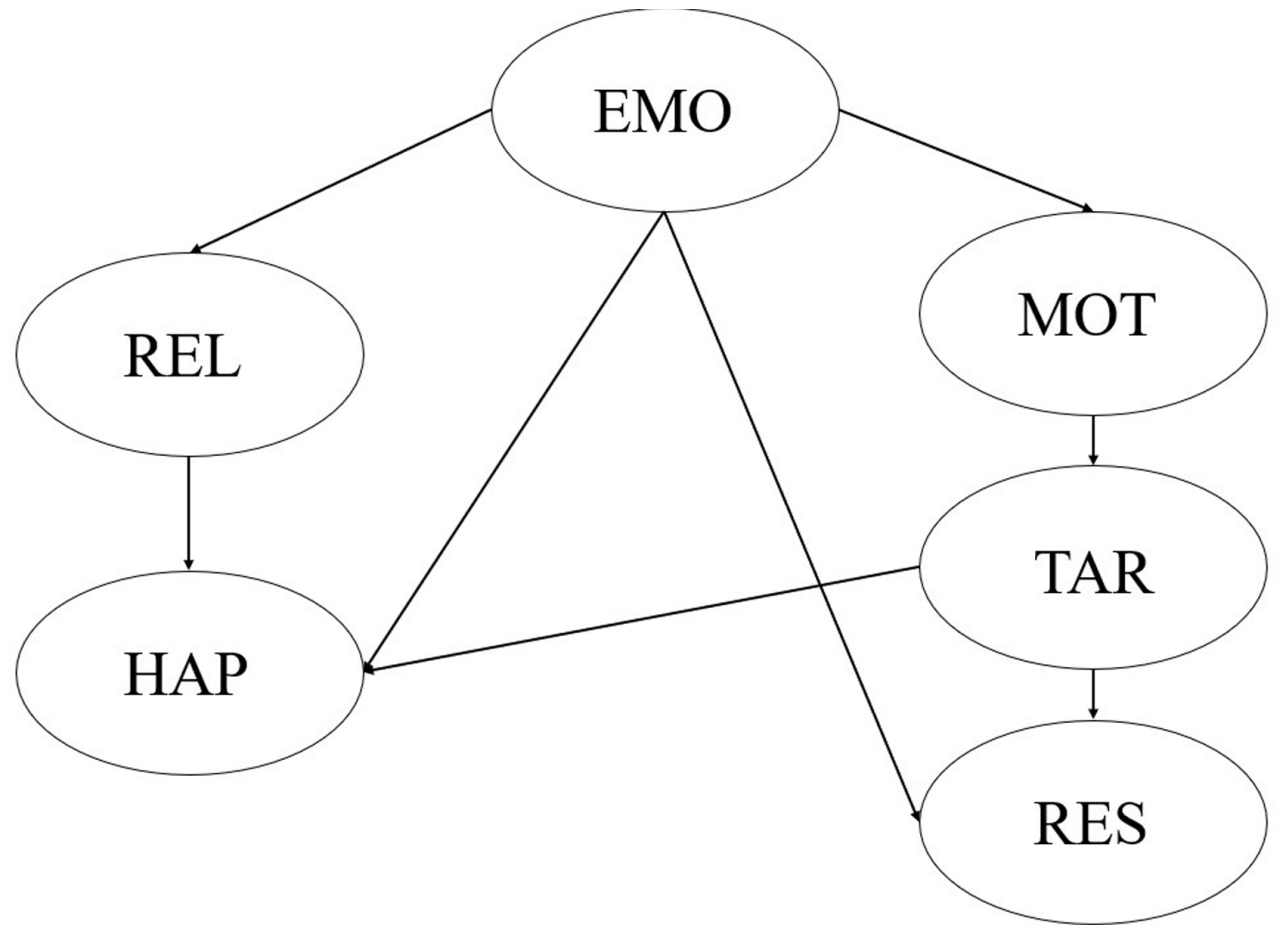
Structural relational model.

This paper intends to construct a structural equation index system composed of five basic dimensional latent variables and 27 observation variables. The relationship between latent variables and observed variables has been shown in Table 1. The basic dimensions of emotion regulation include four observed variables: EMO1, EMO2, EMO3, and EMO4. The basic dimensions of interpersonal relationship include five observed variables: REL1, REL2, REL3, REL4, and REL5. The basic dimensions of happiness consist of five observed variables: HAP1, HAP2, HAP3, HAP4, and HAP5. The basic dimensions of learning motivation consist of five observed variables: MOT1, MOT2, MOT3, MOT4, and MOT5. The basic dimensions of target positioning are composed of four observed variables: TAR1, TAR2, TAR3, and TAR4. The basic dimensions of resilience are composed of four observed variables: RES1, RES2, RES3, and RES4. The theory and former studies assume a direct relationship between emotion regulation, interpersonal relationship, happiness, learning motivation, target positioning, and resilience. The structural relationship model is displayed in Figure 1.

**Table 1:** The relationship between latent variables and observed variables.

Latent variable	Observed variable	Code
Happiness	Life satisfaction	HAP1
		HAP4
	Sense of meaning in life	HAP2
		HAP5
	Life fulfillment	HAP3
Interpersonal Relationship	Opposite-sex interaction	REL1
		REL2
	Communication skills	REL3
		REL4
	Interpersonal trust	REL5
Learning Motivation	Lack of motivation	MOT1
	Possessiveness	MOT2
	Procrastination	MOT3
	Attention	MOT4
	Perseverance	MOT5
Emotion Regulation	Emotion stability	EMO1
		EMO3
	Susceptibility	EMO2
	Emotional control efficacy	EMO4
Target positioning	Oriented life after graduation	TAR1
		TAR2
	Long-term plan for future	TAR3
	Value of life	TAR4
Resilience	Attitude toward challenges	RES1
	Frustrated recovery	RES2
	Emotion toward challenges	RES3
	Coping with challenges	RES4

## Results

### Description statistics and correlations

The description statistics and correlation coefficients among the study are shown in Tables 2, 3. Sixty-eight percent were female (*n* = 621) and 31% were male (*n* = 283) in the available data. The origin from urban areas was about 49% (*n* = 448), while the origin from rural areas was 51% (*n* = 456). There are 326 participants with childhood left-behind experience.

**Table 2:** Description statistics.

Variables	Maximum	Minimum	Median	Quartile	
				Lower quartile	Upper quartile
Gender (1 = male)	2	1	2	1	2
Origin (1 = urban areas)	2	1	2	1	2
Left-behind	2	1	2	1	2
Experience (1 = with left-behind experience)					
Interpersonal relationship	25	5	12	9	14
Emotion regulation	20	4	11	8	13
Happiness	25	5	19	16	21
Resilience	20	4	14	12	16
Learning motivation	25	5	14	11	16
Target positioning	20	4	11	9	13

**Table 3:** Description statistics and correlations.

Variables	Mean	SD	1	2	3	4	5	6	7	8	9
1. Gender (1 = male)	1.69	0.464	1								
2. Origin (1 = urban areas)	1.5	0.5	0.089^**^	1							
3. Left-behind experience (1 = with left-behind experience)	1.64	0.48	−0.03	−0.344^**^	1						
4. Interpersonal	11.79	4.019	0.012	0.153^**^	−0.167^**^	1					
Relationship											
5. Emotion Regulation	10.8	3.431	0.056	0.035	−0.110^**^	0.529^**^	1				
6. Happiness	18.52	3.623	0.026	−0.098^**^	0.098^**^	−0.556^**^	−0.422^**^	1			
7. Resilience	14.26	2.693	−0.087^**^	−0.149^**^	0.084^*^	−0.566^**^	−0.521^**^	0.601^**^	1		
8. Learning Motivation	13.69	4.224	−0.03	0.091^**^	−0.098^**^	0.502^**^	0.535^**^	−0.519^**^	−0.503^**^	1	
9. Target	11.18	3.167	−0.016	0.119^**^	−0.109^**^	0.480^**^	0.410^**^	−0.556^**^	−0.570^**^	0.579^**^	1
Positioning											

Significance level: ^*^p < 0.05; ^**^p < 0.001.

Lambda correlations showed that gender positively correlated with origin (*r* = 0.089, *p* < 0.001). Phi correlations showed that left-behind experience negatively correlated with gender and origin.

Spearman’s correlations displayed that Gender negatively correlated with resilience (*r* = −0.087, *p* < 0.01). Origin positively correlated with interpersonal relationship (*r* = 0.153, *p* < 0.001), target positioning (*r* = 0.119, *p* < 0.001), and learning motivation (*r* = 0.091, *p* < 0.001), but negatively correlated with happiness (*r* = −0.098, *p* < 0.001) and resilience (*r* = −0.149, *p* < 0.001). Left-behind experience negatively correlated with interpersonal relationship (*r* = −0.167, *p* < 0.001), learning motivation (*r* = −0.098, *p* < 0.001), emotion regulation (*r* = −0.110, *p* < 0.001), and target positioning (*r* = −0.109, *p* < 0.001), but positively correlated with resilience (*r* = 0.084, *p* < 0.05) and happiness (*r* = 0.098, *p* < 0.001).

Pearson’s correlations showed that interpersonal relationship positively correlated with emotion regulation (*r* = 0.529, *p* < 0.001), learning motivation (*r* = 0.502, *p* < 0.001), and target positioning (*r* = 0.480, *p* < 0.001), but negatively correlated with happiness (*r* = −0.556, *p* < 0.001) and resilience (*r* = −0.566, *p* < 0.001).

Emotion regulation positively correlated with learning motivation (*r* = 0.535, *p* < 0.001) and target positioning (*r* = 0.410, *p* < 0.001), but negatively correlated with happiness (*r* = −0.422, *p* < 0.001) and resilience (*r* = −0.521, *p* < 0.001).

Happiness positively correlated with resilience (*r* = 0.601, *p* < 0.001), but negatively correlated with learning motivation (*r* = −0.519, *p* < 0.001) and target positioning (*r* = −0.556, *p* < 0.001). Resilience negatively correlated with learning motivation (*r* = −0.503, *p* < 0.001) and target positioning (*r* = −0.570, *p* < 0.001). There was a significant positive correlation between Learning motivation and target positioning (*r* = 0.579, *p* < 0.001).

### Reliability and validity test

The scale was examined for reliability and validity by using SPSS 25.0 software. The Cronbach’s α value of each dimension fluctuates between 0.656 and 0.835, indicating that the reliability of the scale performed relatively well. Besides, the KMO value and Barlett’s sphere test were relatively good as the KMO value of each dimension fluctuated between 0.636 and 0.847, which were all better than 0.6, and the *t*-test values were significant at the 0.05 level which indicated the structural model of performance’s validity was good.

### Model fit test and correlation

The structural equation model was built by operating AMOS 22.0 software and was estimated by using the maximum likelihood method to explore the relationship and action path of the happiness and resilience of university students.

**Figure 2:**
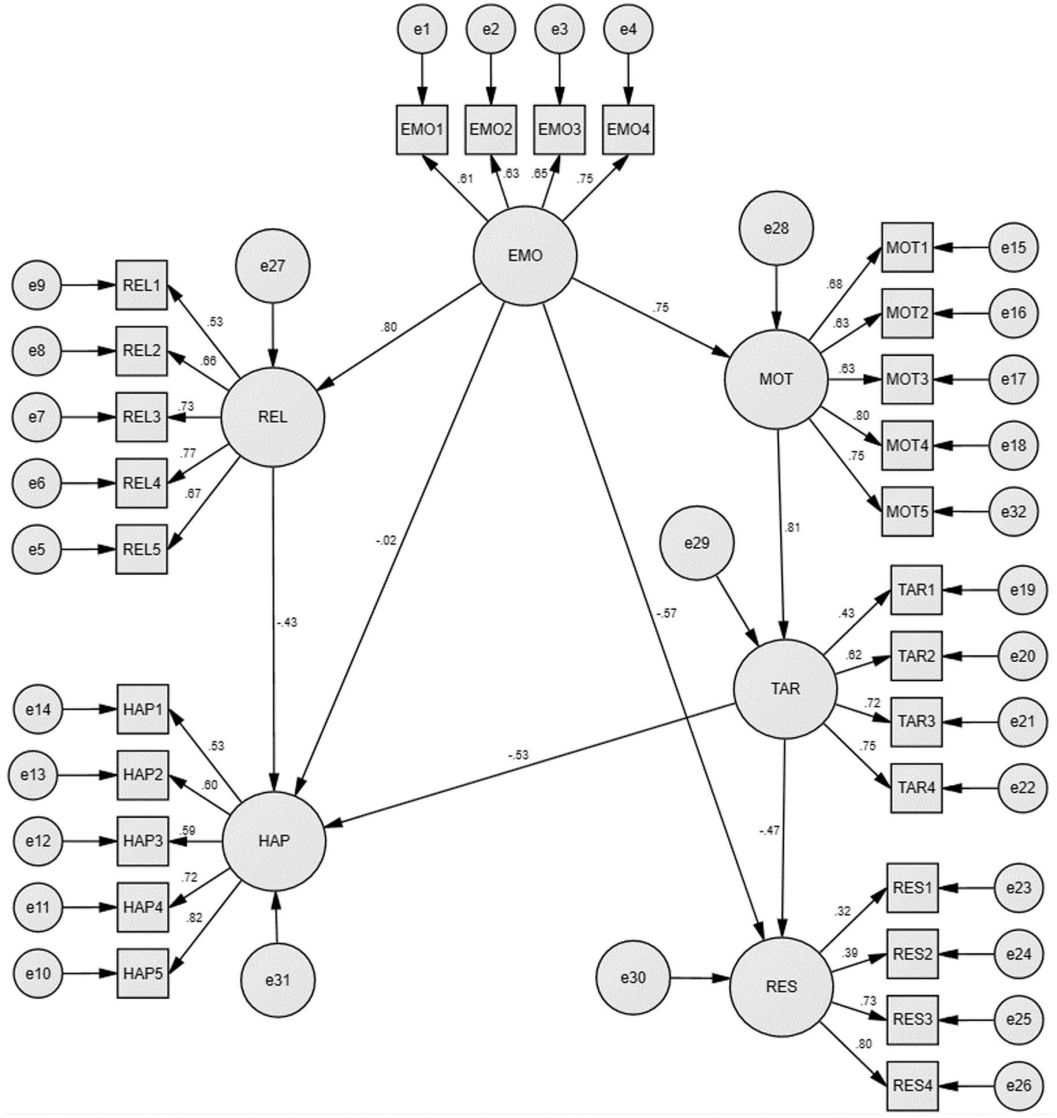
The initial structural equation model.

**Figure 3:**
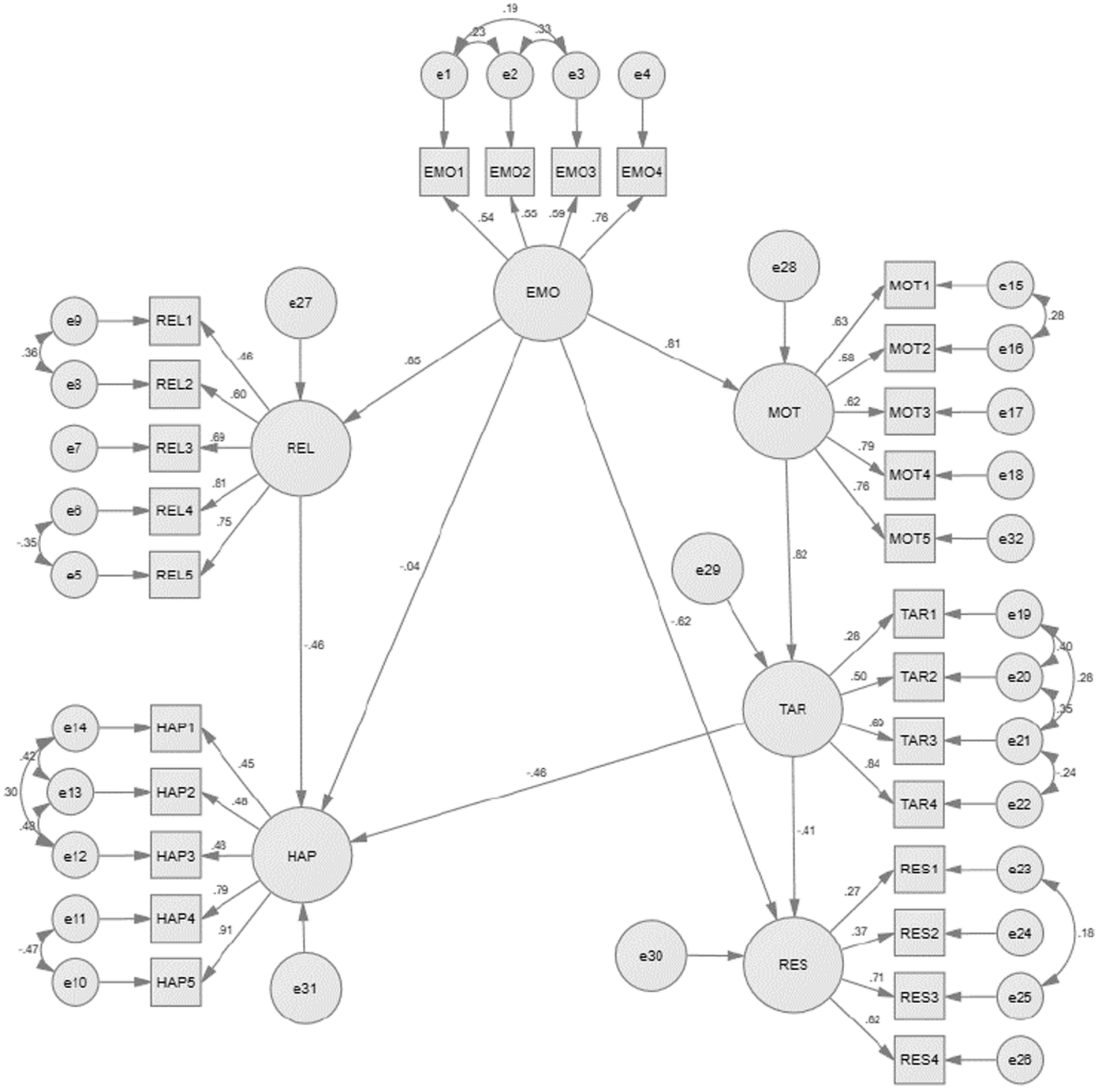
The revised structural equation model.

After simulating the initial model, the correction index MI value among the five latent variables of emotion regulation, interpersonal relationship, happiness, learning motivation, and target positioning was relatively very large. [e8−e9], [15−e16] equal residual paths were added to modify the model. The fitting index is relatively good. Besides, the value of *p* of each path except the path of emotion regulation to happiness after the correction was statistically significant at the level of 0.05. The initial model and the final model are displayed in Figures 2, 3, respectively. The model fit index is shown in Table 4.

**Table 4:** Model fit index.

Fitting index		Initial model	Revised model
Absolute fit index	CMIN/DF	7.86	5.173
	GFI	0.8	0.877
	RMSEA	0.087	0.068
	AGFI	0.761	0.845
Value-added fitting index	NFI	0.786	0.866
	CFI	0.807	0.888
	IFI	0.808	0.889
Simplified fitting index	AIC	2607.62	1710.94
	CAIC	2967.64	2158.06

### Path analysis of the revised model

#### Direct effect

Learned from Table 5, emotion regulation has a positive effect on learning motivation and interpersonal relationship, and each standard path estimate was 0.845 (*p* < 0.001) and 0.915 (*p* < 0.001). But emotion regulation negatively affects resilience with the −0.420 standard path estimate (*p* < 0.001). Learning motivation had a positive effect on target positioning, and the standard path estimate was 0.809 (*p* < 0.001). Interpersonal relationship had a negative effect on happiness, and the standard path estimate was −0.343 (*p* < 0.001). Target positioning had a negative effect on resilience and happiness, and the standard path estimate was −0.612 (*p* < 0.001) and − 0.600 (*p* < 0.001). Emotion regulation had a negative effect on happiness, and the standard path estimate was −0.041, which was not significant (*p* = 0.640).

**Table 5:** The effect relationship between the factors in the fitted model.

Variable			Standard path estimate	C.R.	p
Learning	←	Emotion	0.811	12.8	***
Motivation		Regulation			
Interpersonal	←	Emotion Regulation	0.846	14.174	***
Relationship					
Target	←	Learning	0.824	7.305	***
Positioning		Motivation			
Happiness	←	Interpersonal	−0.457	−6.547	***
		Relationship			
Happiness	←	Emotion	−0.041	−0.468	0.64
		Regulation			
Resilience	←	Target	−0.411	−4.783	***
		Positioning			
Resilience	←	Emotion Regulation	−0.621	−6.404	***
Happiness	←	Target	−0.459	−6.251	***
		Positioning			

Significance level: ^***^p < 0.001.

#### Meditation effect

In this study, the Bootstrap method of deviation-corrected non-parametric percent position confidence interval was used to examine the chain mediating effect and simple mediating effect. The number of repeated random sampling was set to 2,000, and the significance was tested based on whether the 95% CI contained 0.

As shown in the Table 6, emotion regulation had a negative effect on happiness through interpersonal relationship, and the mediating effect was −0.264 (*p* = 0.001). Emotion regulation negatively affected happiness and resilience through learning motivation and target positioning, and the chain mediating effects were − 0.384 (*p* = 0.001) and − 0.391 (*p* = 0.001).

**Table 6:** Analysis of the mediating effect between relationship, emotion regulation, happiness, resilience, and learning motivation.

Intermediary path	Meditation effect	95%CI		Significance
		Lower limit	Upper limit	
Emotion Regulation → Interpersonal Relationship → Happiness	−0.387	−0.517	−0.282	0.001
Emotion Regulation → Learning Motivation → Target positioning → Happiness	−0.307	−0.39	−0.233	0.001
Emotion Regulation → Learning Motivation → Target positioning → Resilience	−0.275	−0.346	−0.207	0.001

## Discussion

This is the first study using a questionnaire to explore the relationship and internal mechanism of emotion regulation, interpersonal relationship, learning motivation, target positioning, happiness, and resilience among 904 students from a university in Sichuan Province. The result showed that learning motivation and target positioning played a chain mediating role in the influence of emotion regulation on happiness and resilience. Besides, interpersonal relationship played an intermediary role in the effect between emotion regulation and happiness. This research helps to further understand the factors that influence happiness and resilience among university students and their internal connections with happiness and resilience. It also gives suggestions to improve happiness and resilience. The specific analysis was as follows.

Learning motivation and target positioning played a chain mediating role in the influence of emotion regulation on happiness and resilience, which is consistent with the hypothesis of this research. Emotion regulation can indirectly predict happiness and resilience by affecting learning motivation and target positioning. Under positive emotions, students are able to understand the learning content from multiple levels and angles, organize and summarize knowledge in an orderly way, and change from mechanistic passive learning to meaningful learning. Whereas negative emotions are the opposite. Individuals with a high level of emotion regulation can adopt adaptive emotion regulation strategies to reduce the influence of negative emotions and generate positive emotions, so as to stimulate higher learning motivation. Motivation can lead to goal-directed behavior. The high level of learning motivation is closely correlated to a high level of learning self-control, a high standard of learning engagement, and high academic achievement, which all contribute to the orientation of university students in their graduation goals and life targets. Individuals who have clear targets are more likely to persist in challenges and difficulties (Schulenberg et al., 2008), and experience more happiness.

Interpersonal relationship played an intermediating role in the influence of emotion regulation on happiness, which is consistent with the hypothesis of this study, and verified the results of the previous study (Ling et al., 2013). Emotion regulation is closely related to interpersonal communication situations. Individuals with high emotion regulation ability can express reasonable emotions and control inappropriate emotional experiences in different interpersonal communication environments, so as to cultivate a good interpersonal atmosphere and form a good interpersonal relationship. A good interpersonal relationship is a key to improving satisfaction and happiness (Okada et al., 2010). Interpersonal resources are an essential source to obtain instrumental assistance and affective support. In the context of Chinese collectivism, interpersonal relationship plays an important role. Good interpersonal relationship predicts the experience of happiness, while bad interpersonal relationship makes university students face greater psychological pressure, thus affecting their happiness.

This study reveals the internal mechanism of emotion regulation on happiness and resilience, and answers how emotion regulation affects happiness and resilience. Additionally, the two chain mediation models displayed in this study have certain implications for improving happiness and resilience: the cultivation of emotion regulation ability is important for the formation of good interpersonal relationships, the promotion of learning motivation, the specific positioning of goals, and the improvement of happiness and resilience. For students, it is necessary to encourage positive learning and open courses on graduation career planning, but more attention should be paid to emotional experience and expression, to improve happiness and psychological resilience. Subsequent research can continue to explore whether (1) different emotion regulation strategies in the same chain-mediated model still significantly predict happiness and resilience, (2) different goal orientations, such as internal and external goals, are still influenced by learning motivation in the same chain-mediated model, and (3) different target positioning plays a mediating role in the relationship between emotional regulation and well-being and resilience. Moreover, longitudinal studies can be designed to test the chain mediating model proposed in this study.

## Conclusion

The cross-sectional study that used data collected between April 2022 and May 2022 examined the chain mediating role of learning motivation and target positioning in the influence of emotion regulation on happiness and resilience, and the mediating role of interpersonal relationship in the effect of emotion regulation on happiness and resilience. The results show that learning motivation and target positioning played a complete chain mediating role in the relationship between emotion regulation and happiness, but played a partially mediating role in the relationship between emotion regulation and resilience. Interpersonal relationship played a dominating mediating role in the influence of emotion regulation on happiness.

## Data availability statement

The raw data supporting the conclusions of this article will be made available by the authors, without undue reservation.

## Ethics statement

The studies involving human participants were reviewed and approved by the local legislation and institutional requirements. The patients/participants provided their written informed consent to participate in this study.

## Author contributions

WL and XC conceived and designed the study, and drafted the method section. XC drafted the rest of the manuscript and contributed equally to this work. ZH extensively instructed and revised the manuscript. All authors contributed to the article and approved the submitted version.

## Funding

The work was supported by the Sichuan College Student Ideological and Political Education Research Center (grant number CSZ20044); Sichuan Applied Psychology Research Center (grant number CSXL-202A07); and College Mathematics Teaching Research and Development Center of Universities (grant number CMC20220403).

## Conflict of interest

The authors declare that the research was conducted in the absence of any commercial or financial relationships that could be construed as a potential conflict of interest.

## Publisher’s note

All claims expressed in this article are solely those of the authors and do not necessarily represent those of their affiliated organizations, or those of the publisher, the editors and the reviewers. Any product that may be evaluated in this article, or claim that may be made by its manufacturer, is not guaranteed or endorsed by the publisher.

## Supplementary material

The Supplementary material for this article can be found online at: https://www.frontiersin.org/articles/10.3389/fpsyg.2022.1029655/full#supplementary-material
