# Supplementary material for: The effect of emotion regulation on happiness and resilience of university students: The chain mediating role of learning motivation and target positioning
Source: Front Psychol. 2022 Dec 9;13:1029655. doi: 10.3389/fpsyg.2022.1029655 (PMC9784224; doi:10.3389/fpsyg.2022.1029655)
Supplement: Supplementary file 1 [file Table_1.DOCX]

Reliability and validity test results

| Latent variable | Observed variable code | Factor loading | Cronbach’s  Coefficient | KMO  value | Barlett’s sphericity test | | |
| --- | --- | --- | --- | --- | --- | --- | --- |
|  |  |  |  |  | Approximate  Chi-square | Degree of freedom | significance |
| Interpersonal  Relationship(REL) | REL1 | 0.691 | 0.808 | 0.818 | 1394.307 | 10 | *** |
|  | REL2 | 0.788 |  |  |  |  |  |
|  | REL3 | 0.793 |  |  |  |  |  |
|  | REL4 | 0.807 |  |  |  |  |  |
|  | REL5 | 0.682 |  |  |  |  |  |
| Emotion  Regulation(EMO) | EMO1 | 0.773 | 0.784 | 0.765 | 989.604 | 6 | *** |
|  | EMO2 | 0.773 |  |  |  |  |  |
|  | EMO3 | 0.799 |  |  |  |  |  |
|  | EMO4 | 0.773 |  |  |  |  |  |
| Happiness(HAP) | HAP1 | 0.734 | 0.815 | 0.789 | 1521.012 | 10 | *** |
|  | HAP2 | 0.798 |  |  |  |  |  |
|  | HAP3 | 0.768 |  |  |  |  |  |
|  | HAP4 | 0.735 |  |  |  |  |  |
|  | HAP5 | 0.759 |  |  |  |  |  |
| Resilience(RES) | RES1 | <0.60 | 0.656 | 0.636 | 603.576 | 6 | *** |
|  | RES2 | 0.622 |  |  |  |  |  |
|  | RES3 | 0.819 |  |  |  |  |  |
|  | RES4 | 0.791 |  |  |  |  |  |
| Learning  Motivation(MOT) | MOT1 | 0.783 | 0.835 | 0.847 | 1576.905 | 10 | *** |
|  | MOT2 | 0.759 |  |  |  |  |  |
|  | MOT3 | 0.744 |  |  |  |  |  |
|  | MOT4 | 0.820 |  |  |  |  |  |
|  | MOT5 | 0.773 |  |  |  |  |  |
| Target  Positioning(TAR) | TAR1 | 0.673 | 0.747 | 0.730 | 866.143 | 6 | *** |
|  | TAR2 | 0.828 |  |  |  |  |  |
|  | TAR3 | 0.824 |  |  |  |  |  |
|  | TAR4 | 0.682 |  |  |  |  |  |
